# Development of FRET-based indicators for visualizing homophilic *trans* interaction of a clustered protocadherin

**DOI:** 10.1038/s41598-021-01481-2

**Published:** 2021-11-15

**Authors:** Takashi Kanadome, Natsumi Hoshino, Takeharu Nagai, Tomoki Matsuda, Takeshi Yagi

**Affiliations:** 1 Department of Biomolecular Science and Engineering, SANKEN (The Institute of Scientific and Industrial Research), Osaka University, 8-1 Mihogaoka, Ibaraki, 567-0047 Japan; 2 KOKORO-Biology Group, Laboratories for Integrated Biology, Graduate School of Frontier Biosciences, Osaka University, Suita, 565-0871 Japan; 3 Japan Science and Technology Agency (JST), Precursory Research for Embryonic Science and Technology (PREST), Kawaguchi, Saitama 332-0012 Japan

**Keywords:** Biological techniques, Biotechnology, Cell biology, Neuroscience

## Abstract

Clustered protocadherins (Pcdhs), which are cell adhesion molecules, play a fundamental role in self-recognition and non-self-discrimination by conferring diversity on the cell surface. Although systematic cell-based aggregation assays provide information regarding the binding properties of Pcdhs, direct visualization of Pcdh *trans* interactions across cells remains challenging. Here, we present Förster resonance energy transfer (FRET)-based indicators for directly visualizing Pcdh *trans* interactions. We developed the indicators by individually inserting FRET donor and acceptor fluorescent proteins (FPs) into the ectodomain of Pcdh molecules. They enabled successful visualization of specific *trans* interactions of Pcdh and revealed that the Pcdh *trans* interaction is highly sensitive to changes in extracellular Ca^2+^ levels. We expect that FRET-based indicators for visualizing Pcdh *trans* interactions will provide a new approach for investigating the roles of Pcdh in self-recognition and non-self-discrimination processes.

## Introduction

Self-recognition and non-self-discrimination among neurons play a fundamental role in the assembly of neural circuits to form neural connections with other neurons but not with themselves. This process is called self-avoidance and is mediated in part by clustered protocadherins (Pcdhs). Pcdh is a cell adhesion molecule; in mice, its 58 isoforms are encoded by the *Pcdhα*, *Pcdhβ*, and *Pcdhγ* gene clusters (14*α*, 22*β*, and 22*γ*), which are tandemly arranged on the same chromosome^1,2^. Individual neurons have been shown to constitutively express five C-type *Pcdh* isoforms (*αC1*, *αC2*, *γC3*, *γC4*, and *γC5*) and stochastically express the remaining 53 isoforms with variable combinations^3–5^. Pcdh shows adhesive activity solely between cells with the same combinatory expression patterns of isoforms^6,7^. Therefore, it is proposed that Pcdh functions as a diversified molecule that confers individualities on cells and that the self is recognized by the homophilic interaction of Pcdh across neurites derived from identical neurons; thereafter, these neurites repel each other by unidentified mechanisms^8–10^. On the basis of experimental evidence, Pcdhγ has been proven to be necessary for the self-avoidance of dendrites in Purkinje cells and retinal starburst amacrine (SAC) cells. Genetic ablation of *Pcdhγ* caused dendrites derived from identical Purkinje cells and retinal SAC cells to be crossed, and functional neural connections were constructed between dendrites of identical retinal SAC cells^11–13^. Notably, a single Pcdh isoform was able to rescue these abnormalities in retinal SAC cells^11,12^. In addition, Pcdh has been observed to be involved in a wide range of biological phenomena, such as axonal coalescence of olfactory sensory neurons^14,15^, axonal projection of serotonergic neurons^16–18^, formation of dendrite branching complexity^19–21^, and synapse maturation^22,23^.

Till date, Pcdh interaction analysis has relied primarily on cell aggregation assays using K562 cells^6,7,24–28^. Because K562 cells lack endogenous cell adhesion molecules and they are observed as separate cells under a microscope, the binding property of the cell adhesion molecules introduced into K562 cells can be evaluated as cell aggregates^29^. This elegant and systematic experimental system has revealed that Pcdh interacts homophilically, and a single mismatched Pcdh is sufficient to interfere with this homophilic interaction^6,7^. However, direct visualization of Pcdh interactions across living cells remains challenging.

Förster resonance energy transfer (FRET) is a process that an excited energy of a donor molecule is nonradiatively transferred to a juxtaposed acceptor molecule via a dipole–dipole resonance interaction^30^. FRET efficiency is mainly determined by spectra overlap, distance, and orientation between donor and acceptor molecules. In life science fields, fluorescent proteins (FPs) have often been used as FRET donor and acceptor molecules. When FRET occurs between donor and acceptor FPs, a decrease in donor fluorescence and an increase in acceptor fluorescence are confirmed. Thereby, FRET is readily detected as the fluorescence ratio between donor fluorescence and acceptor fluorescence by donor excitation. As other methods to detect FRET, fluorescence lifetime imaging of a donor FP and recovery of donor fluorescence by acceptor photobleaching have been employed. On the basis of the principles of FRET, a lot of indicators to detect protein conformation changes and protein–protein interactions have been developed^31^. Of particular note is the indicator for detecting the N-cadherin *trans* interaction across living cells. FRET donor and acceptor FPs are separately inserted into the ectodomain of N-cadherin molecules, which are close to the *trans*-interacting interface^32^. Using this FRET-based indicator for N-cadherin *trans* interaction, Kim et al. investigated the Ca^2+^-responsive dynamics of N-cadherin *trans* interaction across cells^32^.

In this study, we developed indicators for directly visualizing the Pcdh *trans* interaction across cells by separately inserting FRET donor and acceptor FPs into Pcdh molecules, similar to the FRET-based indicator for N-cadherin *trans* interaction. The indicators showed a FRET efficiency of over 25% at the cell adhesion sites. We successfully visualized the Pcdh *trans* interactions across cells using simple ratio imaging. By creating indicators with chimeric Pcdh that do not interact with the original indicators, we confirmed that these indicators specifically visualize the homophilic Pcdh *trans* interaction. We additionally confirmed a Ca^2+^ dependency of Pcdh *trans* interaction, through the FRET ratio change by chelation of extracellular Ca^2+^. These results suggest that the FRET-based Pcdh indicators could serve as a new means for analyzing Pcdh *trans* interactions.

## Results

### Insertion of FPs into Pcdh for development of the FRET-based Pcdh indicators

**Figure 1:**
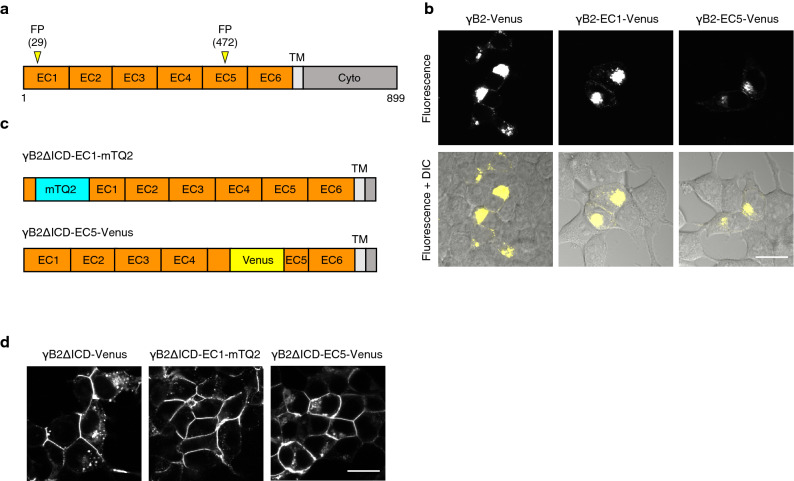
Molecular design and cellular localization of FP-inserted γB2. (**a**) Schematics of full-length protocadherin-γB2 (γB2). γB2 is represented as serially repeated extracellular domains (EC domains), a transmembrane region (TM), and a cytoplasmic region (Cyto). The insertion positions (amino acid position 29th and 472nd residues in the matured form) of fluorescent protein (FP) are indicated by yellow arrowheads. (**b**) The localization of Venus-inserted γB2 constructs in HEK293T cells. HEK293T cells expressing the indicated constructs were observed using a confocal microscope. Fluorescence images (upper) and merged fluorescence and differential interference contrast (DIC) images (lower) are shown. Scale bar, 20 μm. (**c**) Schematics of FP-inserted γB2ΔICDs. In γB2ΔICD-EC1-mTQ2 and γB2ΔICD-EC5-Venus, mTurquoise2 (mTQ2) and Venus are inserted in γB2ΔICD at the positions as indicated in (a). ΔICD denotes deletion of an intracellular domain (ICD), which leads to efficient localization of γB2 at the plasma membrane. (**d**) The localization of FP-inserted γB2ΔICDs in HEK293T cells. HEK293T cells expressing the indicated constructs were observed using a confocal microscope. Scale bar, 20 μm.

To develop FRET-based Pcdh indicators, we used the protocadherin-γB2 (γB2) isoform because structural information was available regarding a *trans*-dimer formed by longer ectodomain fragments (EC1-EC5) of γB2 (PDB: 5T9T)^27^, which was helpful in determining the insertion sites of FPs for the FRET donor and acceptor. We expected that the loop between the βB and βC strands in EC1 of one γB2 molecule and the loop between the βC and βD strands in EC5 of another γB2 molecule would be close to each other with the formation of a *trans*-dimer. On the basis of this expectation, we individually inserted yellow FP Venus into these regions (amino acid position 29th and 472nd residues in the mature form, respectively; Fig. 1a) and examined the effect of FP insertion on the localization of γB2. Venus-inserted γB2s, γB2-EC1-Venus, and γB2-EC5-Venus were mainly localized at the perinuclear region in HEK293T cells, similar to C-terminal Venus-fused γB2 (γB2-Venus) (Fig. 1b). This result indicates that the insertion of Venus into γB2 hardly compromised the localization of γB2. Since the low localization of γB2 at the plasma membrane hampers the development of FRET-based indicators for monitoring γB2 *trans* interactions across cells, we deleted the intracellular domain (ICD) to efficiently localize FP-inserted γB2s at the plasma membrane^33^. We prepared γB2ΔICDs in which cyan FP mTurquoise2 (mTQ2) and Venus were inserted into the EC1 and EC5 domains as the FRET donor and acceptor, respectively (γB2ΔICD-EC1-mTQ2 and γB2ΔICD-EC5-Venus) (Fig. 1c). They were efficiently localized at the plasma membrane similar to γB2ΔICD-Venus (Fig. 1d).

### FRET-based γB2ΔICD indicators can monitor homophilic *trans* interaction of γB2

**Figure 2:**
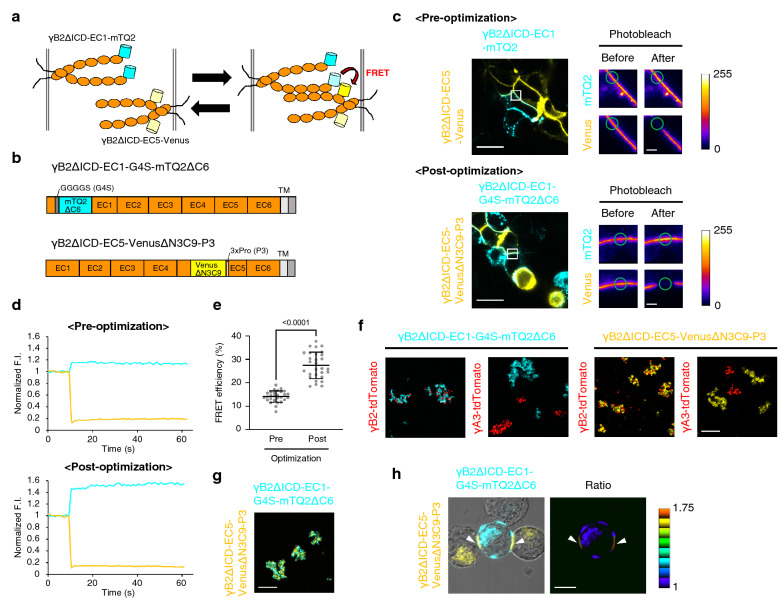
The γB2 *trans* interaction monitored by FRET-based γB2 indicators. (**a**) Schematics of the γB2 *trans* interaction, as visualized using the FRET-based γB2 indicators. The orange ovals represent the EC domains of γB2. mTQ2 and Venus are represented as cyan and yellow cylinders, respectively. (**b**) Schematics of linker-optimized FRET-based γB2 indicators. The C-terminal 6 amino acids of mTQ2 are deleted, and a GGGGS (G4S) linker is inserted at the N-terminus of mTQ2 in γB2ΔICD-EC1-G4S-mTQ2ΔC6. The 3 N-terminal amino acids and the 9 C-terminal amino acids of Venus are deleted, and a 3xPro (P3) linker is inserted at the C-terminus of Venus in γB2ΔICD-EC5-VenusΔN3C9-P3. (**c**) Representative acceptor photobleaching experiments of the pre- and post-optimized FRET-based γB2ΔICD indicators in HEK293T cells. HEK293T cells individually expressing the indicated constructs were co-cultured (left panels). The panels on the right represent magnified fluorescence images of white squared regions in left panels before and after acceptor photobleaching at green circles. The color bars indicate the range of the fluorescence intensities of mTQ2 and Venus (arbitrary units). Scale bars, 20 μm (left panel) and 2 μm (right panel). (**d**) Alterations in fluorescence intensities of mTQ2 and Venus by acceptor photobleaching in (**c**) were followed and plotted over time. (**e**) Mean values of FRET efficiency for the pre- and post-optimized FRET-based γB2ΔICD indicators (Pre, *n* = 28; Post, *n* = 29). Data are shown as the means ± SD. Significant difference was analyzed by Welch’s *t* test. *p* value is described in the graph. (**f**) Binding specificity of the FRET-based γB2ΔICD indicators by cell aggregation assay. K562 cells individually expressing the indicated constructs were co-cultured. Scale bar, 200 μm. (**g**) The interaction between γB2ΔICD-EC1-mTQ2ΔC6 and γB2ΔICD-EC5-VenusΔN3C9-P3, as revealed by cell aggregation assay. K562 cells individually expressing the indicated constructs were co-cultured. Scale bar, 200 μm. (**h**) Direct visualization of the γB2 *trans* interaction using the FRET-based γB2ΔICD indicators. K562 cells individually expressing the indicated constructs were co-cultured. The merged fluorescence and DIC image is shown in the left panel. The ratio image of Venus/mTQ2 is shown as intensity-modulated display (IMD) in the right panel. Scale bar, 10 μm.

To examine whether FRET occurs by γB2 *trans* interaction across cells (Fig. 2a), we co-cultured HEK293T cells individually expressing γB2ΔICD-EC1-mTQ2 and γB2ΔICD-EC5-Venus and assessed FRET by acceptor photobleaching at the cell adhesion sites. In the acceptor photobleaching, Venus in a region of the cell adhesion sites was photobleached using an intense light for Venus excitation and an alteration of mTQ2 fluorescence before and after photobleaching was measured (Fig. 2c,d). Based on the alteration of mTQ2 fluorescence, we calculated FRET efficiency. The average FRET efficiency was 14.1 ± 2.42% (Fig. 2e, mean ± SD, pre-optimization). To increase FRET efficiency, we optimized the linkers between the FPs and γB2. We eventually obtained γB2ΔICD-EC1-G4S-mTQ2ΔC6 and γB2ΔICD-EC5-VenusΔN3C9-P3 as post-optimized FRET-based γB2ΔICD indicators (Fig. 2b, Supplementary Fig. 1). FRET at cell adhesion sites was assessed (Fig. 2c,d), and the average FRET efficiency was 27.4 ± 5.67% (Fig. 2e, mean ± SD, post-optimization), which was approximately twice as high as the pre-optimized FRET efficiency.

A cell aggregation assay using K562 cells in previous studies showed that Pcdh isoforms mediate specific interactions only between identical isoforms^6,7^. To examine the effect of FP-insertion into γB2 on the binding specificity, we performed a cell aggregation assay using K562 cells individually transfected with expression plasmids encoding FRET-based γB2ΔICD indicators and C-terminal tdTomato-fused γB2 (γB2-tdTomato) and γA3 (γA3-tdTomato) isoforms. Both types of cells expressing γB2ΔICD-G4S-mTQ2ΔC6 and γB2ΔICD-EC5-VenusΔN3C9-P3 aggregated with cells expressing γB2-tdTomato; however, they did not aggregate with cells expressing γA3-tdTomato and segregated into homophilic aggregates (Fig. 2f), indicating that FRET-based γB2ΔICD indicators retain homophilic interactions. We observed that γB2ΔICD-EC1-G4S-mTQ2ΔC6 and γB2ΔICD-EC5-VenusΔN3C9-P3 mediate homophilic interactions with each other (Fig. 2g). Finally, we assessed FRET by acquiring a Venus/mTQ2 ratio image of cell aggregates formed by cells individually expressing γB2ΔICD-EC1-G4S-mTQ2ΔC6 and γB2ΔICD-EC5-VenusΔN3C9-P3. We acquired fluorescence images of γB2ΔICD-EC1-G4S-mTQ2ΔC6 and γB2ΔICD-EC5-VenusΔN3C9-P3 by excitation of mTQ2 and composed a ratio image as intensity modulated display (IMD). Consequently, a higher ratio was confirmed at the cell adhesion sites between cells expressing γB2ΔICD-EC1-G4S-mTQ2ΔC6 and γB2ΔICD-EC5-VenusΔN3C9-P3 (Fig. 2h), indicating that the FRET-based γB2ΔICD indicators enable monitoring of Pcdh-mediated cell–cell adhesions as a Venus/mTQ2 ratio readout.

### FRET-based γB2ΔICD indicators detect specific γB2 *trans* interaction

**Figure 3:**
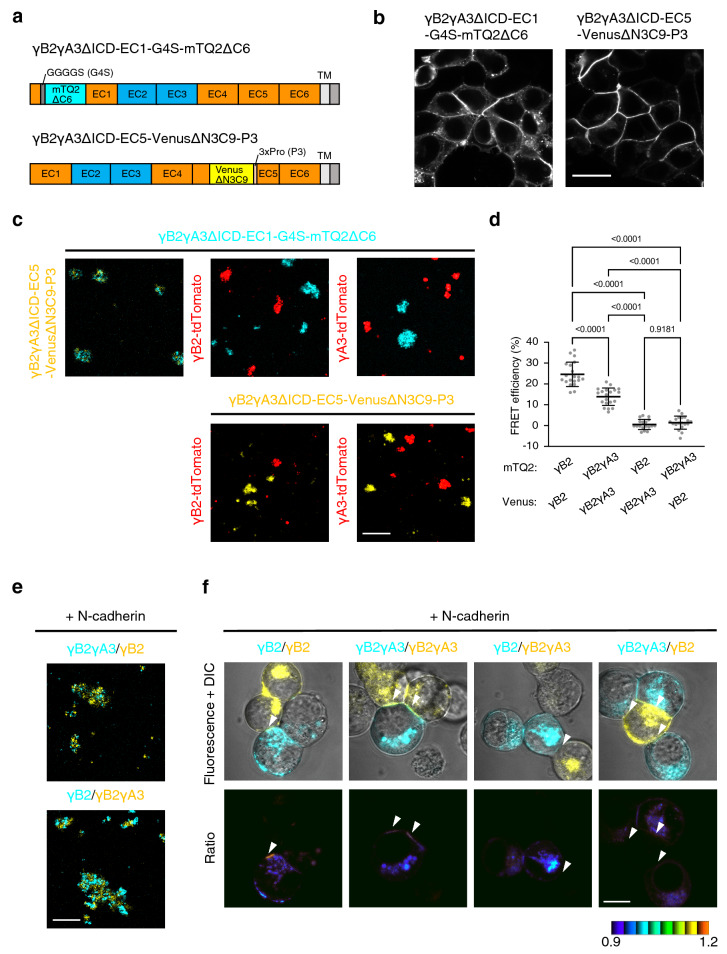
Specificity of FRET-based γB2ΔICD indicators. (**a**) Schematics of FRET-based chimeric γB2γA3ΔICD indicators. The EC2-EC3 domains of the FRET-based γB2ΔICD indicators were swapped by those of γA3. The orange and light blue boxes are the EC domains from γB2 and γA3, respectively. (**b**) The localization of the FRET-based chimeric γB2γA3ΔICD indicators in HEK293T cells. Scale bar, 20 μm. (**c**) Binding specificity of the FRET-based chimeric γB2γA3ΔICD indicators, as revealed by cell aggregation assay. K562 cells individually expressing the indicated constructs were co-cultured. Scale bar, 200 μm. (**d**) Evaluation of the FRET-based chimeric γB2γA3ΔICD indicators and the FRET-based γB2ΔICD indicators by acceptor photobleaching in HEK293T cells. Acceptor photobleaching at the cell adhesion sites was performed using co-cultured HEK293T cells in the indicated combination, and FRET efficiency was calculated [*n* = 20 (γB2–γB2), *n* = 20 (γB2γA3–γB2γA3), *n* = 18 (γB2–γB2γA3), *n* = 19 (γB2γA3–γB2). The combination is described as mTQ2-Venus pair]. Data are shown as the means ± SD. Significant differences were analyzed by Welch’s ANOVA test, followed by Dunnett’s T3 multiple comparison test. *p* values are indicated in the graph. (**e**) Cell aggregation between the FRET-based γB2ΔICD- and chimeric γB2γA3ΔICD indicator-expressing cells, mediated by co-expressing N-cadherin. K562 cells individually expressing the indicated FRET constructs and N-cadherin were co-cultured. Scale bar, 200 μm. (**f**) FRET between the FRET-based γB2ΔICD- and chimeric γB2γA3ΔICD indicators. K562 cells expressing N-cadherin and the FRET-based γB2ΔICD- or the chimeric γB2γA3ΔICD indicators were co-cultured in the indicated combination. The merged fluorescence and DIC images are shown in the upper panels. The ratio images of Venus/mTQ2 are shown as IMD in the lower panels. mTQ2- and Venus-positive cell adhesion sites are indicated by white arrowheads. Scale bar, 10 μm.

To confirm the specificity of the FRET-based γB2ΔICD indicators, we prepared FRET-based indicators to monitor the interaction of other Pcdh isoforms and examined whether FRET occurs between the indicators with γB2ΔICD and other Pcdh. We initially prepared mTQ2- and Venus-inserted γA3ΔICDs with insertion sites corresponding to those of the FRET-based γB2ΔICD indicators. However, almost no FRET was observed between them (data not shown). A previous cell aggregation assay using K562 cells indicated that chimeric Pcdhs whose EC2-EC3 domains were swapped by those of other Pcdh isoforms interact homophilically; however, they did not interact with parental Pcdhs^25^. On the basis of this knowledge, we prepared FRET-based chimeric γB2γA3ΔICD indicators (Fig. 3a). Both γB2γA3ΔICD-EC1-G4S-mTQ2ΔC6 and γB2γA3ΔICD-EC5-VenusΔN3C9-P3 were localized at the cell adhesion sites in HEK293T cells (Fig. 3b). Next, we examined the binding specificity of FRET-based chimeric γB2γA3ΔICD indicators using a cell aggregation assay in K562 cells. While cells individually expressing FRET-based chimeric γB2γA3ΔICD indicators aggregated with each other, they segregated into discrete aggregates with cells expressing γB2-tdTomato or γA3-tdTomato (Fig. 3c). This result indicates that the FRET-based chimeric γB2γA3ΔICD indicators mediate homophilic interactions. To examine the specificity of the FRET-based γB2ΔICD indicators, we individually expressed the FRET-based γB2ΔICD and chimeric γB2γA3ΔICD indicators in HEK293T cells and assessed FRET by acceptor photobleaching at the cell adhesion sites. Although FRET between the same pairs of indicators was confirmed, different pairs did not exhibit FRET (Fig. 3d). We further analyzed the specificity of the indicators in K562 cells as a FRET readout. Since the FRET-based γB2ΔICD and chimeric γB2γA3ΔICD indicators mediate homophilic interactions (Figs. 2f, 3c), the cells expressing them should be segregated. N-cadherin has been reported to bridge K562 cells expressing different Pcdh isoforms^7^. We co-expressed N-cadherin with the FRET-based γB2ΔICD or chimeric γB2γA3ΔICD indicators in K562 cells and co-cultured them. As expected, cell aggregation was confirmed even between the cells expressing a different pair of Pcdh (Fig. 3e), and FRET was confirmed only in the same pairs of indicators at the mTQ2- and Venus-positive cell adhesion sites (Fig. 3f, white arrowheads). These results indicate that the FRET-based γB2ΔICD and chimeric γB2γA3ΔICD indicators specifically monitor γB2 and γB2γA3 *trans* interactions across cells, respectively.

### Ca^2+^dependency of γB2 *trans* interaction revealed by the FRET-based γB2ΔICD indicators

**Figure 4:**
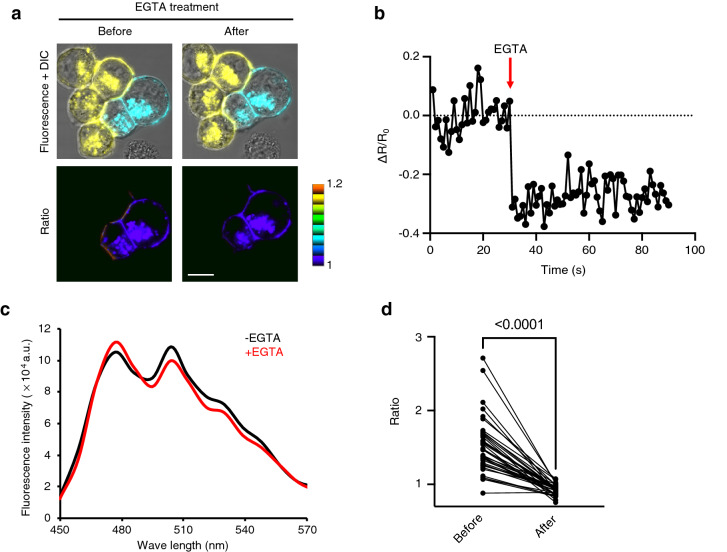
Ca^2+^-dependency of γB2 *trans* interaction. (**a**) The effect of EGTA treatment on the FRET at the cell adhesion sites. K562 cells individually expressing the FRET-based γB2ΔICD indicators were co-cultured. The merged fluorescence and DIC images (upper panels) and the ratio images of Venus/mTQ2 (lower panels) were acquired before and after 5 mM EGTA treatment. Scale bar, 10 μm. (**b**) Change in Venus/mTQ2 ratio (ΔR/R_0_) at a cell adhesion site with EGTA treatment over time. EGTA (5 mM) was added to the medium at 30 s. R_0_ was calculated as the average Venus/mTQ2 ratio value of 30 frames before EGTA treatment. (**c**) Emission spectra before and after EGTA treatment. The emission spectra excited by 405 nm light at the cell adhesion site before and after 5 mM EGTA treatment were acquired. (**d**) Quantitative analysis of ratio changes by EGTA treatment. The FRET ratio values at the cell adhesion sites before and after 5 mM EGTA treatment were measured (*n* = 38). Significant difference was analyzed by Wilcoxon matched-pairs signed rank test. *p* value is indicated in the graph.

Previous reports have examined the Ca^2+^-dependency of Pcdh *trans* interactions in K562 cells, using different approaches, and their conclusions were not consistent^6,25^. To clarify the Ca^2+^-dependency of the Pcdh *trans* interaction, we examined the effect of Ca^2+^chelation by EGTA on the pre-formed cell adhesions mediated by the FRET-based γB2ΔICD indicators in K562 cells. After treatment with EGTA, the Venus/mTQ2 ratio at the cell adhesion sites was reduced (Fig. 4a). Time-lapse imaging showed that a decrease in the Venus/mTQ2 ratio was confirmed within a second (Fig. 4b). Furthermore, emission spectra before and after EGTA treatment indicated that a decrease in the Venus/mTQ2 ratio by Ca^2+^chelation resulted from an increase in the emission of mTQ2, corresponding to a decrease in the emission of Venus (Fig. 4c). Quantitative analysis strongly supported that the Pcdh *trans* interaction was dependent on Ca^2+^ (Fig. 4d).

## Discussion

We have developed FRET-based γB2 indicators, γB2ΔICD-EC1-G4S-mTQ2ΔC6/γB2ΔICD-EC5-VenusΔN3C9-P3, that enabled direct visualization of the Pcdh *trans* interaction and revealed that Pcdh senses changes in extracellular Ca^2+^ levels within a second. The Ca^2+^-dependency of the Pcdh *trans* interaction is controversial. Previously, a cell-based pull-down assay using K562 cells showed that Pcdhγ *trans* interaction is less sensitive to Ca^2+^^6^. In contrast, Rubinstein et al. concluded that Pcdhs mediate cell–cell interactions in a Ca^2+^-dependent manner on the basis of a cell aggregation assay using K562 cells^25^; consistent with these observations, our result for the FRET-based γB2ΔICD in K562 cells clearly demonstrated that the Pcdh *trans* interaction is dependent on Ca^2+^ (Fig. 4).

To show the specificity of the FRET-based γB2ΔICD indicators, we first prepared γA3ΔICD in which FRET donor and acceptor FPs were individually inserted at the corresponding sites to γB2. However, we could not confirm FRET at the cell adhesion sites formed by the cells individually expressing them (data not shown). A previous report showed that dimer structures formed by the EC1-EC5 domains of γB2 and EC1-EC4 domains of γB7 are closely related^27^. In contrast, root mean square deviations (RMSDs) between a dimer structure formed by EC1-EC4 of γA1 and dimer structures formed by EC1-EC5 domains of γB2 and EC1-EC4 domains of γB7 were relatively large^27^. These notions suggest that the overall structures of *trans* dimers between the γA and γB subfamilies are varied. In addition, considering that the FRET phenomenon is sensitive to the distance and orientation of the donor and acceptor FPs, the FRET of FP-inserted γA3 was not detected because of the improper relative orientation. Crystal structures of *trans* dimers formed by longer ectodomains will enable further development of individual indicators for other Pcdh isoforms.

The Pcdh *trans* interaction was analyzed using a cell aggregation assay in K562 cells lacking endogenous cell adhesion molecules. Since this method relies on the observation of cell aggregates, we cannot strictly distinguish the Pcdh *trans* interaction from the other factors that promote cell aggregation. A previous report^7^ and our results (Fig. 3e) showed that cells individually expressing different Pcdh isoforms co-aggregated in the presence of N-cadherin, indicating that homophilic Pcdh *trans* interactions cannot be confirmed as cell aggregates in the presence of other cell adhesion molecules. In contrast, the FRET-based γB2ΔICD indicators visualized homophilic Pcdh *trans* interactions, even in the presence of N-cadherin (Fig. 3f). Furthermore, merged images of fluorescence and DIC in Fig. 4a showed that cell–cell contacts were sustained even after EGTA treatment. However, the ratio images indicated that the Pcdh *trans* interaction was diminished by extracellular Ca^2+^ chelation, suggesting that the FRET-based gB2ΔICD indicators can detect Pcdh *trans* interaction independent of visual cell aggregation.

Cell–cell interactions have been visualized using a bimolecular fluorescence complementation (BiFC) technique^34–39^. Although BiFC-based indicators are potent tools for visualizing cell–cell interactions, they have limitations. The complementation reaction is irreversible, which hampers the monitoring of the dissociation of cell–cell interactions. In contrast, since FRET is reversible, we successfully monitored the dissociation of Pcdh *trans* interactions across cells using the FRET-based gB2ΔICD indicators (Fig. 4a).

We have accomplished only the development of FRET-based γB2 indicators and observation of the Pcdh *trans* interaction across cells by introducing indicators in heterologous cells such as K562 and HEK293T cells. One of the most important issues to be addressed is determination of the spatio-temporal roles of the Pcdh *trans* interaction in biological phenomena such as dendritic self-avoidance. For examination of self-avoidance in neurons, interactions between dendrites derived from identical neurons should be analyzed. In such a case, the FRET-based γB2 indicators must be expressed in the same cells. On the basis of this theory, to examine whether co-expressed FRET-based γB2ΔICD indicators monitor the Pcdh *trans* interaction across cells, we co-cultured K562 cells co-expressing the indicators. However, FRET was hardly detected at not only cell adhesion sites but also non-cell adhesion sites, implying that neither *trans*- nor *cis*-interactions could be visualized between cells co-expressing FRET-based γB2ΔICD indicators (Supplementary Fig. 2). Therefore, we concluded that co-expressed FRET-based γB2ΔICD indicators do not work. One method to avoid this problem is analysis of cell–cell contact sites between two different neurons lacking endogenous Pcdhs. We previously showed that γA3-Venus and γA3-tdTomato co-localized at the process-process contact sites formed by *Pcdhαβγ*-deficient neurons individually expressing them while they localized at the processes as discrete puncta in wild-type neurons^40^. Furthermore, Kostadinov and Sanes revealed that retinal SAC cells expressing a single γC3 isoform showed a reduction in intercellular functional connections, which was considered as self-avoidance between cells^12^. These previous findings suggest that combinatorially expressed endogenous Pcdhs prevent an exogenous Pcdh isoform from forming a *trans* interaction; therefore, an exogenous Pcdh isoform can interact homophilically across processes of two different neurons lacking endogenous Pcdhs. Pcdh *trans* interactions could be visualized in the self-avoidance process using *Pcdhαβγ*-deficient neurons individually expressing the FRET-based γB2 indicators. In this study, we used FRET-based γB2ΔICDs which lack an ICD to efficiently localize at the plasma membrane. However, an ICD has been proven to be essential for self-recognition and non-self-discrimination processes in olfactory sensory neurons^15^. Therefore, FRET-based γB2 indicators with an ICD must be used in neurons. As a next step, we firstly plan to examine whether the FRET-based γB2 indicators can visualize γB2 *trans*-interaction between *Pcdhαβγ*-deficient neurons. We expect that the FRET-based γB2 indicators would be a start for unveiling the spatio-temporal roles of Pcdh *trans* interactions in self-recognition and non-self-discrimination in neurons as a future effort.

## Methods

### Plasmid construction

All expression plasmids were subcloned into the pCX vector. Full-length mouse Pcdhs C-terminally fused with fluorescent proteins were generated as described previously^38^. We used monomeric Venus (A206K) for the following constructions and described them as Venus. The amino acid positions of Pcdh are described in the immature form in this section. To create Venus-inserted γB2s, γB2-EC1-Venus, and γB2-EC5-Venus, we inserted Venus flanked by NheI sites at amino acid position 58 in the EC1 domain and 501 in the EC5 of γB2 C-terminally fused with the PA tag^41^ by overlapping PCR. γB2ΔICD constructs were created by the deletion of ICD (739–940 amino acids). To generate γB2ΔICD constructs inserted with linker-modified FPs for FRET imaging, we inserted PCR products encoding linker-modified FPs flanked by NheI sites into γB2ΔICD constructs C-terminally fused with the PA tag. For the construction of γB2γA3ΔICD chimeric constructs, an EC1 domain containing a propeptide (1–58 amino acids), EC4-EC6 domains containing a transmembrane region, and a part of the cytoplasmic region (343–940 amino acids) from γB2, and EC2-EC3 domains (127–340 amino acids) from γA3 were connected using an overlapping PCR.

### Cell culture and plasmid transfection

HEK293T cells (RIKEN BRC) were maintained in Dulbecco’s modified Eagle’s medium (DMEM, Sigma-Aldrich) supplemented with 10% (v/v) fetal bovine serum (FBS, Biowest) at 37 °C in humidified air containing 5% CO_2_. K562 cells (RIKEN BRC) were maintained in Iscove’s modified Dulbecco’s medium (IMDM, FUJIFILM) supplemented with 10% (v/v) FBS (Thermo Fisher Scientific) and 50 μg/mL kanamycin at 37 °C in humidified air containing 5% CO_2_. HEK293T cells were transfected using polyethylenimine “MAX” (Cosmo Bio). K562 cells were electroporated using an Amaxa 4D-Nucleofector (Lonza).

### Cell imaging

HEK293T cells grown on glass-bottomed dishes coated with Cellmatrix Type I-C (Nitta gelatin) were transiently transfected with expression plasmids and incubated overnight. Before imaging, the medium was replaced with phenol red-free DMEM/F12 (Thermo Fisher Scientific). Fluorescence and DIC images were acquired using an Olympus FV-1000 laser scanning confocal microscope with an IX81 microscope equipped with a × 60, 1.35 numerical aperture (NA) oil-immersion objective lens (UPLSAPO60XO) (Olympus). For co-culture experiments, HEK293T cells were individually transfected with expression plasmids and incubated overnight. The cells were detached from dishes using trypsin–EDTA (0.25%) and phenol red (Thermo Fisher Scientific) and mixed in 10% FBS/DMEM. Imaging was performed after 24 h.

Transfected K562 cells were rotated at 30 rpm overnight at 37 °C in humidified air containing 5% CO_2_. Before imaging, the cells were decanted into glass-bottomed dishes coated with 0.1% (w/v) polyethylenimine (Sigma-Aldrich) for 1 h at 37 °C under humidified air containing 5% CO_2._ The images were acquired using an LSM780 confocal microscope equipped with a Plan-Apochromat × 10, 0.45 NA objective lens (for observation of cell aggregates), and a Plan-Apochromat × 63, 1.40 NA oil immersion objective lens (for ratio imaging and spectrum imaging) (Carl Zeiss).

For ratio imaging, mTQ2 was excited with a 405-nm laser. The donor and acceptor emissions were collected at 463–500 nm and 520–620 nm, respectively. IMD images were created using the MetaMorph software (Molecular Devices).

For spectrum imaging, mTQ2 was excited with a 405-nm laser. Emission was collected over a spectrum with a width of 8.9 nm.

### Acceptor photobleaching

Photobleaching was performed as previously described^42,43^. mTQ2 and Venus were excited using a 405 nm laser diode and the 515 nm line of a multi-argon laser, and their emissions were collected using bandpass filters set at 460–500 nm and 530–630 nm, respectively. The image size was set to 512 × 512 pixels. The pinhole diameter was set to 300 μm. For each time-lapse series, an optical zoom of 3.0 was used, and images were acquired without intervals at 200 × 50 pixels. Venus signals at the cell adhesion sites were photobleached using the main scanner in tornado scan mode with the 515 nm line of a multi-argon laser set at 100% transmission on one frame. The bleached region was set as a circle with a diameter of 20 pixels. The fluorescence intensities within the bleached region and cell-free area (background) were measured using the Olympus FV10-ASW software. After background subtraction, the fluorescence intensities of mTQ2 and Venus in the bleached region were normalized to the mean fluorescence intensity of 10 frames before photobleaching and plotted over time. The mean fluorescence intensities of mTQ2 from 10 frames before and after photobleaching were calculated, and the FRET efficiency was calculated using Eq. (1).

1 $$FRET \; efficiency \left(\%\right)=1-\frac{F_{BB}}{F_{AB}} \times 100 \; \left(\%\right),$$

where *F*_*BB*_ and *F*_*AB*_ are the fluorescence intensities of mTQ2 before and after photobleaching, respectively.

## Supplementary Information

Supplementary Information.

## Data Availability

The data and materials supporting this research are available from the authors upon request.
