# Supplementary material for: Development of FRET-based indicators for visualizing homophilic trans interaction of a clustered protocadherin
Source: Sci Rep. 2021 Nov 15;11:22237. doi: 10.1038/s41598-021-01481-2 (PMC8593154; doi:10.1038/s41598-021-01481-2)
Supplement: Supplementary file 1 — Supplementary Information. [file 41598_2021_1481_MOESM1_ESM.docx]

**Supplementary information**

**Development of FRET-based indicators for visualizing homophilic *trans* interaction of a clustered protocadherin**

Takashi Kanadome^1,3,4^, Natsumi Hoshino^2,4^ , Takeharu Nagai^1^, Tomoki Matsuda^1*^, and Takeshi Yagi^2*^

^1^ Department of Biomolecular Science and Engineering, SANKEN (The Institute of Scientific and Industrial Research), Osaka University, 8-1 Mihogaoka, Ibaraki 567-0047, Japan

^2^ KOKORO-Biology Group, Laboratories for Integrated Biology, Graduate School of Frontier Biosciences, Osaka University Suita, 565-0871, Japan

^3^ Japan Science and Technology Agency (JST), Precursory Research for Embryonic Science and Technology (PREST), Kawaguchi, Saitama, 332-0012, Japan

^4^ Co-first authors

^*^Correspondence: tmatsuda@sanken.osaka-u.ac.jp (T.M.) and yagi@fbs.osaka-u.ac.jp (T.Y.)

**Supplementary Figure 1.** FRET-based γB2ΔICD indicators on linker optimization process. **(a)** A series of mTQ2-inserted γB2ΔICDs **(b)** A series of Venus-inserted γB2ΔICDs. **(c)** Acceptor photobleaching at the cell adhesion sites was performed using co-cultured HEK293T cells in the indicated combination, and FRET efficiency was calculated [*n* = 28 (mTQ2/Venus), *n* = 24 (G4S-mTQ2ΔC6/Venus), *n* = 24 (G4S-mTQ2ΔC6/VenusΔN3C9), *n* = 14 (G4S-mTQ2ΔC6/P3-VenusΔN3C9), *n* = 14 (G4S-mTQ2ΔC6/P6-VenusΔN3C9), *n* = 14 (G4S-mTQ2ΔC6/P9-VenusΔN3C9), *n* = 10 (G4S-mTQ2ΔC6/VenusΔN3C9-P2), *n* = 29 (G4S-mTQ2ΔC6/VenusΔN3C9-P3), *n* = 10 (G4S-mTQ2ΔC6/VenusΔN3C9-P4), *n* = 14 (G4S-mTQ2ΔC6/VenusΔN3C9-P6), *n* = 14 (G4S-mTQ2ΔC6/VenusΔN3C9-P9)]. Data are shown as the means ± SD. mTQ2/Venus and G4S-mTQ2ΔC6/VenusΔN3C9-P3 pairs correspond to the Pre and Post in Fig. 2e, respectively.

**Supplementary Figure 2.** FRET between cells co-expressing the FRET-based γB2ΔICD indicators. **(a)** FRET of individually expressed (left) or co-expressed (right) FRET-based γB2ΔICD indicators in K562 cells. Scale bar, 10 μm. **(b)** Comparison of FRET ratio between individually expressed and co-expressed FRET-based γB2ΔICD indicators (Individually expressed, *n* = 30; Co-expressed, *n* = 39). FRET ratio at the cell adhesion sites was measured. Data are shown as the means ± SD. Significant difference was analyzed by Welch’s t test. *p* value is described in the graph. **(c)** Comparison of FRET ratio between cell adhesion sites and non-cell adhesion sites of co-expressed FRET-based γB2ΔICD indicators (Cell adhesion site, *n* = 39; Non-cell adhesion site, *n* = 11). Data are shown as the means ± SD. Significant difference was analyzed by Welch’s t test. *p* value is described in the graph.
